# Trabectedin Plus Radiotherapy for Advanced Soft-Tissue Sarcoma: Experience in Forty Patients Treated at a Sarcoma Reference Center

**DOI:** 10.3390/cancers12123740

**Published:** 2020-12-12

**Authors:** Nadia Hindi, Irene Carrasco García, Alberto Sánchez-Camacho, Antonio Gutierrez, Javier Peinado, Inmaculada Rincón, Johanna Benedetti, Pilar Sancho, Paloma Santos, Paloma Sánchez-Bustos, David Marcilla, Victor Encinas, Sara Chacon, Cristobal Muñoz-Casares, David Moura, Javier Martin-Broto

**Affiliations:** 1 Medical Oncology Department, Hospital Universitario Virgen del Rocio, Av Manuel Siurot s/n, 41013 Sevilla, Spain; nhindi@mustbesevilla.org (N.H.); irene.carrasco.sspa@juntadeandalucia.es (I.C.G.); alberto.sanchezcamacho.sspa@juntadeandalucia.es (A.S.-C.); johana.benedetti.sspa@juntadeandalucia.es (J.B.); mariap.sancho.sspa@juntadeandalucia.es (P.S.); paloma.santos.sspa@juntadeandalucia.es (P.S.); 2 TERABIS Group, IBiS (Instituto de Biomedicina de Sevilla), 41013 Sevilla, Spain; pbustos-ibis@us.es (P.S.-B.); david.moura@usal.es (D.M.); 3 Hematology Department, University Hospital Son Espases, 07120 Mallorca, Spain; antoniom.gutierrez@ssib.es; 4 Radiation Oncology Department, University Hospital Virgen del Rocio, 41013 Sevilla, Spain; javier.peinado.sspa@juntadeandalucia.es (J.P.); inmaculada.rincon.sspa@juntadeandalucia.es (I.R.); 5 Biología Molecular del Cáncer, IBiS (Instituto de Biomedicina de Sevilla), 41013 Sevilla, Spain; 6 Centro de Investigación Biomédica en Red de Cáncer (CIBERONC), 28029 Madrid, Spain; 7 Pathology Department, Hospital Universitario Virgen del Rocio, Av Manuel Siurot s/n, 41013 Sevilla, Spain; david.marcilla.sspa@juntadeandalucia.es; 8 Musculoskeletal Unit, Radiology Department, Hospital Universitario Virgen del Rocio, Av Manuel Siurot s/n, 41013 Sevilla, Spain; victorm.encinas.sspa@juntadeandalucia.es; 9 Musculoskeletal Tumor Unit, Orthopedics Surgery Department, Hospital Universitario Virgen del Rocio, Av Manuel Siurot s/n, 41013 Sevilla, Spain; sara.chacon.sspa@juntadeandalucia.es; 10 Surgery Department, Hospital Universitario Virgen del Rocio, Av Manuel Siurot s/n, 41013 Sevilla, Spain; franciscoc.munoz.sspa@juntadeandalucia.es

**Keywords:** advanced soft-tissue sarcoma, trabectedin plus radiotherapy, palliative therapy, growth-modulation index

## Abstract

**Simple Summary:**

Active therapeutic options in advanced sarcomas, able to induce durable objective responses, are scarce beyond first line. New strategies for disease and symptomatic control are thus needed. Our aim was to analyze the activity of the combination of trabectedin and palliative radiotherapy in the real-life setting, in patients with pretreated metastatic sarcoma. Our findings on 40 pretreated metastatic soft-tissue sarcoma patients, in terms of objective responses (overall response rate by RECIST of 32.5%) and outcome (median progression-free survival of 7.5 months and median overall survival of 23.5 months), confirm the activity of this regimen, which is a valuable option to consider, especially in patients in which a dimensional response could help for symptomatic control.

**Abstract:**

Symptomatic control and tumoral shrinkage is an unmet need in advanced soft-tissue sarcoma (STS) patients beyond first-line. The combination of trabectedin and radiotherapy showed activity in a recently reported clinical trial in this setting. This retrospective series aims to analyze our experience with the same regimen in the real-life setting. We retrospectively reviewed advanced sarcoma patients treated with trabectedin concomitantly with radiotherapy with palliative intent. Growth-modulation index (GMI) was calculated as a surrogate of efficacy. Forty metastatic patients were analyzed. According to RECIST, there was one (2.5%) complete response, 12 (30%) partial responses, 18 (45%) disease stabilizations, and nine (22.5%) progressions. After a median follow-up of 15 months (range 2–38), median progression-free survival (PFS) and overall survival (OS) were 7.5 months (95% CI 2.8–12.2) and 23.5 months (95% CI 1.1–45.8), respectively. Median GMI was 1.42 (range 0.19–23.76), and in 16 (53%) patients, it was >1.33. In patients with GMI >1.33, median OS was significantly longer than in those with GMI 0–1.33 (median OS 52.1 months (95% CI not reached) vs. 8.9 months (95% CI 6.3–11.6), *p* = 0.028). The combination of trabectedin plus radiotherapy is an active therapeutic option in patients with advanced STS, especially when tumor shrinkage for symptomatic relief is needed.

## 1. Introduction

Soft-tissue sarcoma (STS) is an heterogeneous group of diseases accounting for 1–2% of adult malignant neoplasms [1]. Approximately 20% of patients at diagnosis, and about 30–40% of patients with an initial diagnosis of localized sarcoma will develop distant metastasis and will eventually succumb to the disease. Although it has not been as widely explored as other neoplasms, patients with advanced sarcoma are frequently symptomatic in relation to the disease, with pain and dyspnea being two of the most reported symptoms [2]. Furthermore, the symptom burden increases during the course of the disease: while 50% and 20% of patients experience pain and dyspnea in first line, these numbers rise to 82% and 40%, respectively, in second-line in a series of advanced sarcoma patients [3]. The aim of systemic therapy in patients with advanced STS is mainly disease and symptomatic control, with a small proportion of selected patients in which long-term disease control can be obtained with multimodal therapy including surgery [4]. Patients receiving anthracycline-based combinations in first line, when compared with anthracycline in monotherapy, have a higher chance of achieving a dimensional response, although these combination regimens have failed to show an improvement in terms of overall survival (OS) [5]. Beyond first-line, disease control is modest, with median progression-free survival (PFS) ranging 2.6–4.6 months and expected overall response rate (ORR) according to RECIST 1.1 lower than 10% with currently available options [6,7,8]. This fact, and considering the context of a more symptomatic population, highlights the need in second and further lines of STS of therapeutic options that are able to induce durable disease control and tumor shrinkage. One of these strategies could be the combination of trabectedin and radiotherapy. Trabectedin is a marine-derived alkaloid, approved for the treatment of advanced STS after anthracyclines and ifosfamide. Several mechanisms of action have been described for this drug such as the interference with DNA transcription (as a consequence of its binding to the minor groove of DNA), its ability to induce cell cycle arrest [9], and its effects on tumoral microenvironment, targeting macrophages [10]. Preclinical data showed that trabectedin had a radiosensitizing effect on several cell lines [11,12,13,14].

We have recently reported the results of the combination of trabectedin and low-dose radiotherapy (RT) (30 Gy in 10 fractions) in advanced STS within the international Phase I/II TRASTS trial. In the phase II part of this study, patients received trabectedin 1.5 mg/m^2^ in 24-h continuous infusion with RT, which was started within one hour after completion of the first trabectedin cycle (cycle 1, day 2). The combination showed a manageable toxicity profile and resulted in a highly effective scheme in metastatic STS patients, with 60% of patients achieving RECIST 1.1 responses, according to a central radiological review among the 25 evaluable patients. The median progression-free survival (PFS) in this study exceeded nine months and, interestingly, the 6-month PFS rate was 75%, all of which are remarkable data for second-line therapy in advanced STS [15]. Here, we present our experience in real-life patients with advanced sarcoma treated at our center with the same combination of trabectedin plus radiotherapy.

## 2. Results

Forty consecutive patients were treated and included in this analysis. Gender was balanced and the median age at the time of therapy initiation was 57 years (range 16–80). The main patient characteristics are depicted in Table 1.

Patients diagnosed with L-sarcomas (liposarcoma or leiomyosarcoma) represented 62.5% of the series. All patients had metastatic disease at the start of trabectedin, with a median metastasis-free interval from diagnosis of 16 months (0–268). In 22 patients (55%), one or more symptoms were present at baseline and registered in medical files: pain in 18 cases, mass effect with stiffness or compression in four, dyspnea in two. Thirty-eight patients (95%) had received previous systemic therapy, with a median of previous lines of one (0–6). In eight patients, trabectedin was their first line of systemic therapy for advanced disease: two patients had contraindication to anthracycline and ifosfamide due to advanced age and cardiovascular risk factors, and the other six patients had received anthracycline and ifosfamide in the perioperative setting. Six of the patients (15%) in the current series had previously received trabectedin: three patients achieved RECIST partial responses during the TRASTS trial and subsequently progressed due to new lesions. At progression, trabectedin was maintained off study and RT was administered to the progressive lesions. Another three patients had previously received trabectedin with benefit: one of the patients discontinued therapy in response because he underwent local therapy (isolated limb perfusion) after diagnosis of a local relapse; another patient discontinued therapy while in response due to toxicity (fatigue, nausea, and catheter infection), and the third patient was referred to our center at the time of focal peritoneal progression. In all six pretreated cases, there was previous evidence of benefit from trabectedin and the new lesions were unique or few (oligoprogression), amenable for RT, and symptomatic or rapidly progressive (and judged as potentially symptomatic in a short period). All patients in the series received at least one cycle of trabectedin, with a median of nine cycles (range 2–45) for all the series. Nineteen patients (47.5%) started RT during the first cycle of trabectedin, while 21 (52.5%) received RT later on during therapy. In 27 patients (67.5%) RT was started in the 24 h after the completion of a cycle of trabectedin (in day +2 of the cycle), while in 13 patients RT had a more delayed start with regard to trabectedin administration (from day +3 of the cycle and beyond, being the median of delay of five days). All patients received the complete planned dose of RT. Twenty-eight (70%) received 30 Gy in 10 fractions, and 12 patients received another regimen (see Appendix A) with a median dose of 30 Gy (18–50 Gy). The median number of irradiated lesions was 1 (range 1–2), with 10 patients receiving RT on 2 different target lesions (within the same course of RT). Median size of the biggest irradiated lesion was 59 mm (range 11–218). In five patients (12.5%), the target irradiated lesion was the primary tumor, in 35 patients, the irradiated lesions were metastases, located in the lungs in 12 (30%) patients, the abdominal cavity or retroperitoneum in 12 (30%) patients, and in soft tissue or bones in 12 patients (30%). At the time of the current analysis (24 July 2020), nine patients were still receiving trabectedin, while 31 had withdrawn from therapy: 24 due to radiological progression, four due to clinical progression, two due to death secondary to septic shock (central venous catheter infection and neutropenic fever) without evidence of disease progression, and one due to cardiological problems not related with therapy. After trabectedin plus RT, 24 patients received additional systemic therapy, with a median of one further line (range 1–5). Fourteen patients (35%) experienced a Grade 3 or higher adverse event, the most frequent toxicity being G3–4 neutropenia, occurring in six patients (15%). Two patients (5%) died while in therapy with trabectedin (several months after the concomitant therapy with RT) due to sepsis: one in the context of neutropenic fever after cycle 4 of trabectedin and the other due to non-neutropenic central catheter infection after cycle 16 of trabectedin. More details on toxicity are given in Table 2.

**Figure 1:**
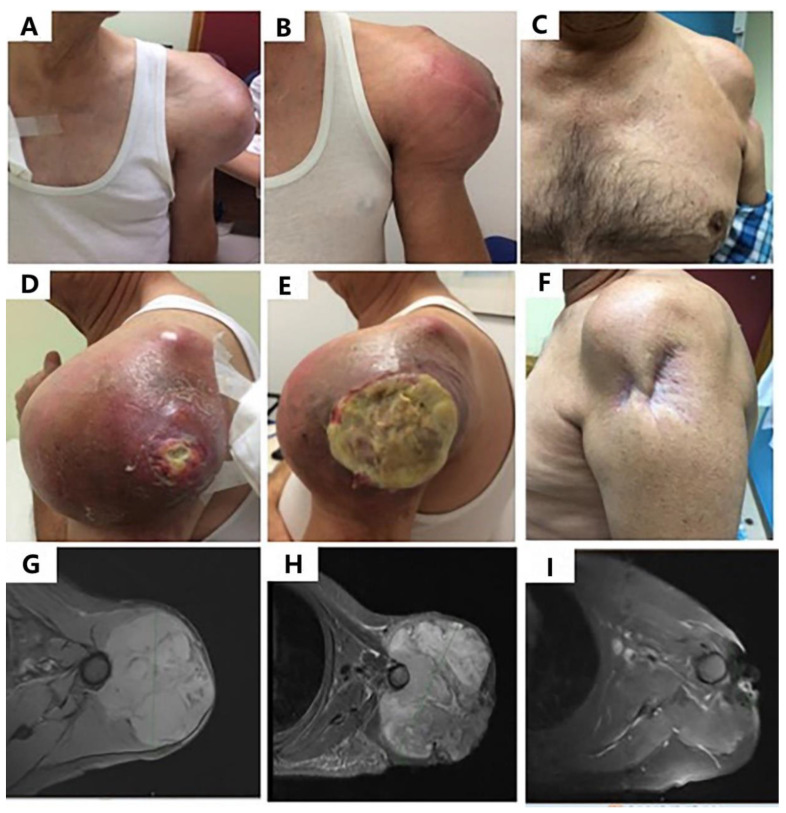
Clinical and RECIST partial response in a 59-year-old man with a metastatic leiomyosarcoma. The patient received trabectedin plus RT (30 Gy in 10 fractions) after rapid progression to doxorubicin-olaratumab. (**A**,**D**) Baseline study (November 2018); (**B**,**E**) Skin ulceration after Cycle 1 of trabectedin and RT (Dec 2018); (**C**,**F**) Long-lasting partial response (September 2019). (**G**–**I**) MRI images. (**G**) Baseline to previous line (September 2018); (**H**) Baseline to trabectedin-RT (November 2018); (**I**) Partial response (August 2019).

**Figure 2:**
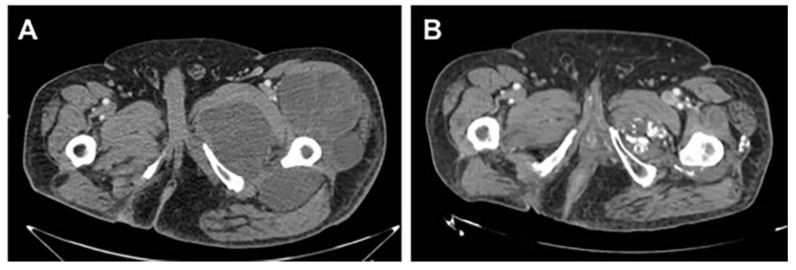
RECIST partial response in a 73-year-old man with a round-cell myxoid liposarcoma. The patient received trabectedin plus RT (39 Gy in 13 fractions) in first line, as he had contraindication to anthracyclines and ifosfamide due to cardiac problems. (**A**) Baseline study (April 2017). (**B**) Long-lasting partial response (June 2019).

According to RECIST 1.1, one (2.5%) patient achieved a complete response (CR), 12 (30%) patients achieved a partial response (PR), 18 (45%) patients obtained stable disease (SD), and nine (22.5%) progressed (PD) as their best response, resulting in an overall response rate (ORR) of 32.5%. Considering only irradiated lesions, there was one (2.5%) CR, 14 (35%) PR, 22 (55%) SD (in 12 of these 22 patients there was some shrinkage in the lesion), and three (7.5%) PD. Examples of partial responses are seen in Figure 1 and Figure 2.

**Figure 3:**
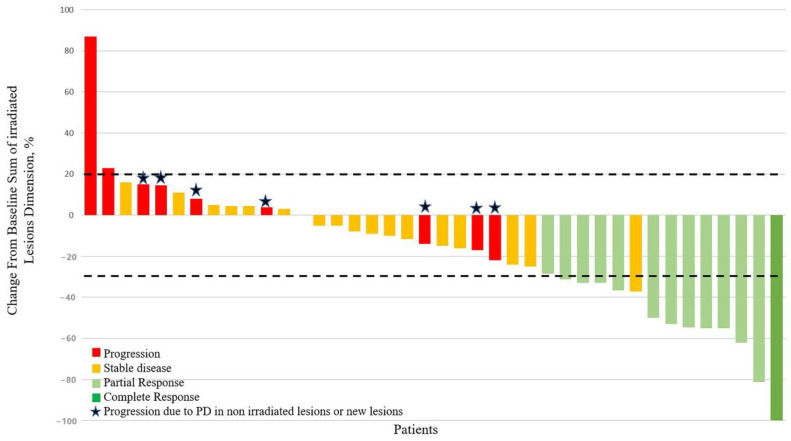
Waterfall plot of the irradiated lesions. Plot shows dimensional change in irradiated lesions as a percentage of the baseline measurement. Lower and upper dashed lines represent the cut-offs for progressive disease (20% increase in the sum of diameters of lesions) and for partial response (30% decrease in the sum of diameters of target lesions), respectively. The colors show the RECIST 1.1 response for all of the disease (including irradiated and non-irradiated lesions). Stars represent patients experiencing disease progression due to new lesions or due to growth in non-irradiated lesions.

The median percentage of shrinkage in the irradiated lesions was −14% (range −100/+87). Figure 3.

Regarding those six patients pre-treated with trabectedin, one patient achieved a PR, four patients remained SD and one progressed. When analyzing responses in irradiated lesions according to the timing of RT, there was one CR and 11 PR among the 27 patients who started RT in the 24 h after administration of trabectedin (ORR 44%), while there were three PR among the 13 patients (23%) who received RT on a more delayed basis. With regard to clinical benefit, among the 22 patients with baseline registered symptoms, in 14 of them (64%), a relief or clinical improvement with trabectedin and RT was clearly recorded in clinical files.

**Figure 4:**
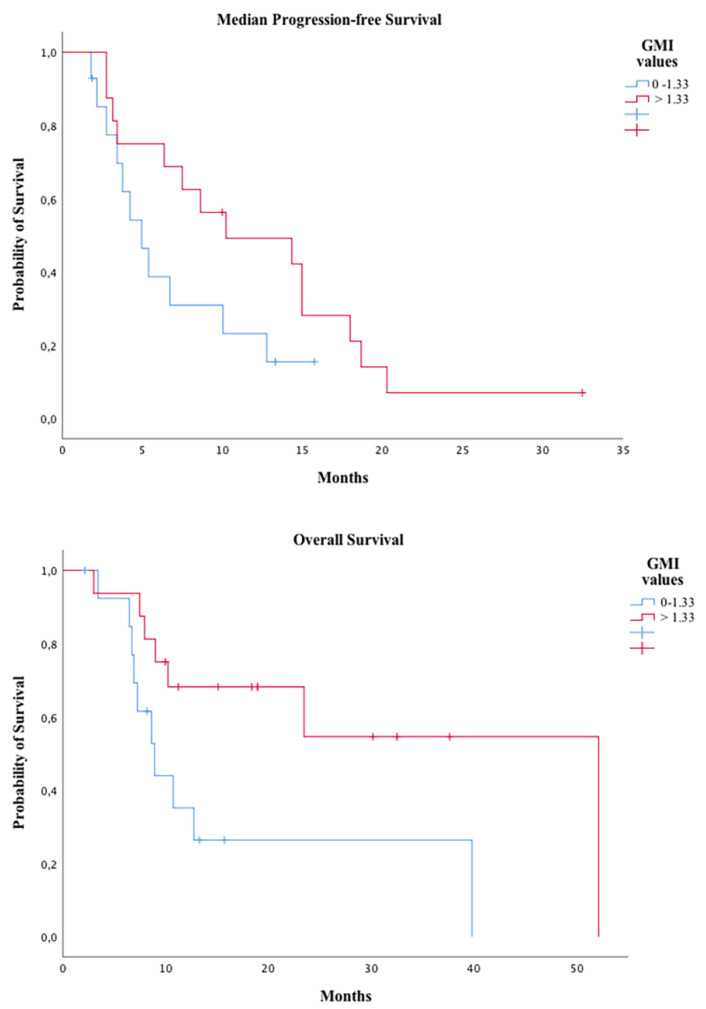
Progression-free survival and overall survival for trabectedin according to growth-modulation index (GMI).

With a median follow-up in the 18 alive patients of 15 months (range 2–38) from the start of trabectedin, 28 patients have progressed, with a median PFS of 7.5 months (95% CI 2.8–12.2), and 22 patients have died, with a median OS of 23.5 months (95% CI 1.1–45.8), since trabectedin initiation. In the univariate analysis, those patients achieving an objective response (CR + PR) to therapy had a longer PFS when compared with those achieving SD (9.6 months (95% CI 2.5-16.6) vs. 4.9 months (95% CI 1.9–7.9) *p* < 0.001) (see Appendix A). Similarly, there was a trend toward a longer median OS for responding patients (43 months (95% CI NA) vs. 6.5 months (95% CI 0.4–12.7), *p* = 0.19. Considering those patients treated immediately before trabectedin with other systemic therapy for advanced disease (*n* = 30), we observed that median PFS for trabectedin (7.5 months (95% CI 3.5–11.5) was more than double the PFS for the previous line (median PFS 3.1 months (95% CI 2.1–4)). The median of growth modulation index (GMI) was 1.42 (range 0.19–23.76) for all 30 patients, being >1.33 in 16 (53%) patients. In those patients with GMI >1.33, the median OS for trabectedin was significantly longer than in those patients with GMI 0–1.33 (median OS 52.1 months (95% CI not reached) vs. 8.9 months (95% CI 6.3–11.6), *p* = 0.028). With regard to PFS, those patients with longer GMI showed a trend toward a better median PFS when compared to those with GMI 0–1.33 (median PFS from trabectedin 10.2 months (95% CI 0.1–20.3) vs. five months (95% CI 3.1–6.9) (*p* = 0.093)), as seen in Figure 4.

## 3. Discussion

Here, we report on a retrospective series of 40 patients with advanced soft-tissue sarcoma treated, in real-life, with the combination of trabectedin plus radiotherapy in a sarcoma reference center. The results in this series are in line with the previous published study considering the median PFS [15], and clearly exceed those expected for trabectedin alone, or other approved second line drugs for sarcoma [6,7,8]. It is important to highlight that these results were obtained with trabectedin and external beam conventional RT in a population with bulky (median size of the irradiated lesion exceeded 5 cm), pretreated, and symptomatic disease. This combination was feasible, and manageable following the tissue constraints for RT. The toxicity profile was similar to that previously reported with trabectedin alone [16]. The median GMI of 1.42 also supports the activity of the combination, as in more than half of the patients, the disease control obtained by trabectedin plus RT was better than in their previous line. These are remarkable results, which favorably compare with previous analysis of GMI for second lines in advanced sarcoma. The analysis of GMI in patients included in phase II trials with trabectedin [17], trabectedin in the real-life population [18], or second lines in general in advanced STS [19] found GMI >1.33 in 29–38.8% of cases. In our series, this proportion of patients was markedly higher (53%) and, as occurred in these analyses, those patients with GMI >1.33 showed a better outcome. It is difficult to elucidate if these differences are exclusively attributable to the addition of RT, but otherwise, the population included in these analyses and our series are similar in terms of the previous number of lines and sarcoma subtypes.

In our previous study, 60% of patients achieved radiological responses, while in the present series, the ORR according to RECIST was 32.5%. This overall response rate is still remarkable, being similar to the most active regimens in advanced STS used in frontline therapy, which is the combination of anthracyclines and ifosfamide [5], and higher than other active combinations in further lines of STS such as gemcitabine-based regimens [20,21,22] or other approved drugs such as eribulin and pazopanib. A potential explanation of the lower response rate observed in the present series with respect to the clinical trial is the lower adherence to the concomitant schedule of therapy. In the previous clinical trial [15], all patients started RT immediately after the complexion of the first cycle of trabectedin. In this real-life series, a third of the patients started RT more than 24 h after trabectedin, and this delay in the initiation of RT could have impacted on the activity of the combination. Indeed, although non-significant due to low numbers, the ORR in the irradiated lesions of those patients who received trabectedin and RT with the concomitant schedule was 44%, while those patients with a delayed administration of RT achieved an ORR of 23%. One of the potential mechanisms of radiosensitization of trabectedin is the induction of G2/M accumulation [15], but this phenomenon seems to be time-dependent, with a peak in the hours immediately after exposure to trabectedin [11], suggesting that the timing of the administration of RT would be relevant. Given the preclinical data and the results in our series, we would advise administering RT as soon as possible after the end of trabectedin administration.

In addition, in the current series, several regimens of radiotherapy were administered. However, this latter fact did not seem to explain the differences in ORR as we did not observe a linear relationship between the RT dose and the responses. Another factor that could also explain some of the observed differences, both in terms of ORR and PFS, is the fact that six of the patients had already been treated with trabectedin, and the median PFS obtained with trabectedin and RT for this group was 3.4 months (95% IC 1–12.7), with only one patient achieving a PR.

Palliative radiotherapy is frequently administered for symptomatic relief [23]. In the small number of documented series with palliative external conventional RT in advanced sarcoma patients, pain improvement is reported in 70–80% of patients [24,25]. In these retrospective series, several RT schedules were used (39 Gy in 13 fractions in one series and up to 25 different regimens in the other one) and radiological responses were not reported, making it challenging to draw comparisons with our findings. However, the data that were reported about the duration of response and survival in these series seem to be inferior to ours. In our series, symptomatic improvement was retrospectively collected from clinical files, which could underestimate our findings, but in two thirds of initially symptomatic patients, a clinical improvement was evident.

The combination of systemic therapy and palliative RT in order to maximize efficacy has not been assessed in sarcoma beyond our trial, with the exception of case reports or small series [26,27]. In other neoplasms, the strategy of adding chemotherapy to low-dose palliative RT with synergistic intention has been tested, with more clinical responses, although a better outcome could not always be demonstrated [28,29,30]. Symptomatic control remains an unmet need in advanced sarcoma patients, needing a better prospective assessment in clinical trials with the proper tools [31]. With the aim of better exploring the activity of the combination of trabectedin and radiotherapy, with a special focus on quality of life assessments, a new prospective multicohort clinical trial is planned in advanced sarcoma with this combination.

## 4. Materials and Methods

### 4.1. Patients

Patients diagnosed with advanced soft-tissue sarcoma and treated with trabectedin in combination with radiotherapy, outside of clinical trial, between April 2015 and March 2020 in the Sarcoma Unit of Virgen del Rocio University Hospital were retrospectively reviewed. Pathological diagnosis was confirmed by expert sarcoma pathologists at our site. In all cases, the possibility of curative surgery had been discussed and ruled out by the multidisciplinary tumor board (MDT). Trabectedin was administered in indication and all patients accepted therapy and signed informed consent for chemotherapy and radiotherapy as per institutional guidelines. Patients included in this analysis were not candidates for the TRASTS trial at the time of the therapy initiation. This research has been approved by local Ethics Committee, with ethic code 1839-N-20.

### 4.2. Treatment

Trabectedin was administered at doses of 1.2–1.5mg/m^2^ through a venous central catheter in 24-h continuous infusion every 21 days in an outpatient setting. All patients received premedication with dexamethasone 4 mg 24 h and 12 h before the start of the trabectedin infusion, and dexamethasone 20 mg IV just before the start of the infusion, in line with the drug brochure. All patients received trabectedin until progression, intolerance, or patient/physician decision. Previous therapy with trabectedin was allowed. In those cases, only data from the time of concomitant therapy was considered for outcome analysis. Radiotherapy was administered with palliative intention, concomitantly with trabectedin. 3 dimensional radiotherapy (3DRT), Intensity-modulated radiation therapy (IMRT), and Volumetric Modulated Arc Therapy (VMAT) techniques were employed using VMAT and IMRT in those cases where targets were close to or in contact with organs at risk. Stereotactic body radiation therapy (SBRT) was not allowed for this protocol. The largest lesions, those which were more symptomatic or those rapidly progressing (even if asymptomatic at that moment, in order to prevent local symptoms), were irradiated, but it was not mandatory to include all the disease sites under the RT fields. Whenever it was not possible to start RT from the first cycle of trabectedin, it was started with the next cycle, aiming to start RT the day after starting a cycle of trabectedin, in order to maximize its synergistic effects. The preferred regimen was 30 Gy in 10 fractions, although other regimens were allowed according to the characteristics of the patient (see more details on RT planification in Appendix B).

### 4.3. Clinical and Radiologic Assessments

Clinical examination, symptoms, blood count, and biochemistry were evaluated at baseline and then monitored every cycle during therapy. Toxicity was recorded based on CTC 5.0. A baseline computed tomography (CT) scan showing disease progression was mandatory. The CT was repeated every three cycles of therapy, as per institutional guidelines. Radiological responses according to RECIST 1.1 [32] were calculated after every CT scan, both in the irradiated lesion and globally for all of the disease (considering as target lesions both irradiated and not irradiated lesions).

### 4.4. Statistical Analysis

Overall response rate (ORR) was calculated as the proportion of patients who achieved a partial or complete RECIST response during therapy. Progression-free survival (PFS) and overall survival (OS) were calculated from the date of the first cycle of trabectedin and estimated by the Kaplan–Meier method. For PFS, the event was considered at the time of radiological evidence of progression or death by any cause, whichever occurred first. In the event of starting any other therapy (including a new course of RT), patients were censored for PFS. For OS, the event was recorded at the date of the last contact. Growth modulation index (GMI) was calculated in those patients with previous systemic therapy for advanced disease as a surrogate of activity of trabectedin plus RT. The GMI was calculated by dividing the time to progression for trabectedin by the time to progression for the immediately previous line. A GMI >1.33 is considered as a sign of activity [17]. Statistical analysis was performed with SPSS version 26.0.

## 5. Conclusions

The combination of trabectedin plus radiotherapy is a highly active therapeutic option in patients with advanced soft-tissue sarcoma. Our real-life series support the results of the already reported Phase I/II trial, as we report here a median PFS longer than seven months. This combination is a worthwhile option, especially in patients requiring tumor shrinkage for symptomatic relief. Whenever possible, the treatment should be administered with the closest sequence possible between the end of the trabectedin infusion to the initiation of radiotherapy, in order to maximize its efficacy.

## Figures and Tables

**Table 1:** Demographics and clinical characteristics of the 40 patients.

**Characteristic**	***n* (%)**
Age at therapy, median (range)	57 (16–80)
Gender Male Female	20 (50) 20 (50)
Histologic subtype Leiomyosarcoma Myxoid liposarcoma Dedifferentiated liposarcoma Synovial MPNST UPS Sarcoma NOS Myxofibrosarcoma High-grade ESS FS-DFSP Pleomorphic rhabdomyosarcoma	18 (45) 3 (7.5) 4 (10) 4 (10) 3 (7.5) 2 (5) 2 (5) 1 (2.5) 1 (2.5) 1 (2.5) 1 (2.5)
Grade 1 2 3	3 (7%) 8 (20%) 29 (72%)
Stage at diagnosis Localized Metastatic	32 (80) 8 (20)
Metastasis-free interval, months (range)	16 (0–268)
Number of previous lines of therapy, median (range)	1 (0–6)

MPSNT: malignant peripheral sheath nerve tumor; sarcoma NOS: not otherwise specified; UPS: undifferentiated pleomorphic sarcoma; ESS: endometrial stromal sarcoma, FS-DFSP: fibrosarcoma- dermatofibrosarcoma protuberans.

**Table 2:** Toxicity Grades 3–5 reported in the series.

Adverse Event	*n* (%)
Hematological toxicity	
Anemia	1 (2.5)
Thrombocytopenia	1 (2.5)
Neutropenia	6 (15)
Neutropenic fever *	2 (5)
Non-Hematological toxicity	
Nausea/vomiting	2 (5)
Creatinine increase	2 (5)
Catheter infection *	1 (2.5)
Trabectedin extravasation	1 (2.5)
Cardiac congestive failure **	1 (2.5)

* Fatal event (Grade 5) in one case of neutropenic fever; ** Patient previously treated with anthracyclines, reversible ejection fraction decrease in the context of supraventricular tachycardia.

## Appendix A

**Table A1:** RT regimens administered.

Regimen	*n* (%)
30 Gy in 10 fractions	28 (70)
39 Gy in 13 fractions	7 (17.5)
45 Gy in 15 fractions	1 (2.5)
45 Gy in 25 fractions	1 (2.5)
50 Gy in 25 fractions	1 (2.5)
20 Gy in 5 fractions	1 (2.5)
18 Gy in 3 fractions	1 (2.5)

**Table A2:** Univariate analysis for PFS and OS.

Factor	Median PFS (95% CI)	*p*	Median OS (95% CI)	*p*
MFI: 0–16 >16	4.3 (0–8.9) 12.8 (5.1–20.4)	0.19	23.5 (2.8–44.2) 37 (2–72.1)	0.79
Metastatic at diagnosis: Yes No	3.4 (0–22.1) 7.5 (3.1–11.9)	0.29	NR 16.5 (0–33.6)	0.078
Age: 0–54 >54	10.2 (0–22.9) 6.8 (5.3–8.3)	0.97	36.2 (12.8–59.7) 12.8 (0–33.9)	0.15
Age: 0–60 >60	10.2 (0.2–20.3) 6.7 (4–9.4)	0.55	36.2 (11.1–61.3) 12.8 (0–34.6)	0.23
Sex: Male Female	7.5 (1.6–13.4) 6.8 (1.6–13.4)	0.43	10.2 (0.4–20) 36.2 (9–20)	0.049
Grade: 1 2 3	10.2 (0.63–19.8) 6.4 (0.4–12.4) 8.6 (3.5–13.8)	0.9	10.2 (NA) 8.6 (7.2–10.1) 32.7 (7.8–57.7)	0.68
Histology: Myxoid liposarcoma Liposarcoma (Other than myxoid) Uterine leiomyosarcoma (LMS) LMS other Other	15 (0–32.1) 7.5 14.5 (11.8–17.1) 6.7 (0–19.3) 5.4 (2.8–8)	0.3	32.7 (0–73.5) 7.5 (NA) 36.2 (NA)NR 9 (8.4–9.6)	0.44
Previous lines: 0–1 >1	6.7 (4.8–8.7) 10 (6.8–13.2)	0.68	32.7 (0–66.4) 23.5 (3.8–43.1)	0.95
Response to trabectedin (*): CR/PR SD PD	9.6 (2.5–16.6) 6.8 (1.9–7.9) 4.1 (0–3)	<0.001	43 (NA) 6.5 (0.4–12.7) 9.3 (1.6–17)	0.19

MFI: metastatic-free interval; * Landmark analysis.

## Appendix B

Radiotherapy treatment details:

A planning CT scan (Toshiba Aquillion^®^, Minato, Tokyo, Japan) with 3–5 mm-thick slices was performed. Treatment volumes were contoured according to ICRU 83, generating a gross tumor volume (GTV), clinical target volume (CTV), and planning target volume (PTV) (and internal target volume (ITV) when possible) considering the TRASTS contouring protocol (see below).

Radiotherapy treatment with 3D conformal radiotherapy or intensity modulated radiotherapy (IMRT) was prescribed to cover 95% of the planning target volume (PTV), which included the gross tumor volume (GTV), plus the antero-posterior and lateral margins of 1.5 cm and superior and inferior margins of 2 cm. If using an internal tumor volume (ITV) method, the margins can be reduced to 0.7 and 1 cm, respectively. Specific normal tissue constraints were: V16 of lungs <20%; V30 of heart <50%; V30 of esophagus <50%; and a maximum dose of 36 Gy for spinal cord. Minor and major deviations were defined in accordance with the planning target volumes and normal tissue constraints. Radiotherapy was administered only in weekdays from Monday to Friday.
