# Erratum: Hindi, N., et al. Trabectedin Plus Radiotherapy for Advanced Soft-Tissue Sarcoma: Experience in Forty Patients Treated at a Sarcoma Reference Center. *Cancers* 2020, *12*, 3740

**DOI:** 10.3390/cancers13071557

**Published:** 2021-03-29

**Authors:** Nadia Hindi, Irene Carrasco García, Alberto Sánchez-Camacho, Antonio Gutierrez, Javier Peinado, Inmaculada Rincón, Johanna Benedetti, Pilar Sancho, Paloma Santos, Paloma Sánchez-Bustos, David Marcilla, Victor Encinas, Sara Chacon, Cristobal Muñoz-Casares, David Moura, Javier Martin-Broto

**Affiliations:** 1 Medical Oncology Department, Hospital Universitario Virgen del Rocio, Av Manuel Siurot s/n, 41013 Sevilla, Spain; nhindi@mustbesevilla.org (N.H.); irene.carrasco.sspa@juntadeandalucia.es (I.C.G.); alberto.sanchezcamacho.sspa@juntadeandalucia.es (A.S.-C.); johana.benedetti.sspa@juntadeandalucia.es (J.B.); mariap.sancho.sspa@juntadeandalucia.es (P.S.); paloma.santos.sspa@juntadeandalucia.es (P.S.); 2 TERABIS Group, IBiS (Instituto de Biomedicina de Sevilla, HUVR, CSIC, US), 41013 Sevilla, Spain; pbustos-ibis@us.es (P.S.-B.); david.moura@usal.es (D.M.); 3 Hematology Department, University Hospital Son Espases, 07120 Mallorca, Spain; antoniom.gutierrez@ssib.es; 4 Radiation Oncology Department, University Hospital Virgen del Rocio, 41013 Sevilla, Spain; javier.peinado.sspa@juntadeandalucia.es (J.P.); inmaculada.rincon.sspa@juntadeandalucia.es (I.R.); 5 Biología Molecular del Cáncer, IBiS (Instituto de Biomedicina de Sevilla), 41013 Sevilla, Spain; 6 Centro de Investigación Biomédica en Red de Cáncer (CIBERONC), 28029 Madrid, Spain; 7 Pathology Department, Hospital Universitario Virgen del Rocio, Av Manuel Siurot s/n, 41013 Sevilla, Spain; david.marcilla.sspa@juntadeandalucia.es; 8 Musculoskeletal Unit, Radiology Department, Hospital Universitario Virgen del Rocio, Av Manuel Siurot s/n, 41013 Sevilla, Spain; victorm.encinas.sspa@juntadeandalucia.es; 9 Musculoskeletal Tumor Unit, Orthopedics Surgery Department, Hospital Universitario Virgen del Rocio, Av Manuel Siurot s/n, 41013 Sevilla, Spain; sara.chacon.sspa@juntadeandalucia.es; 10 Surgery Department, Hospital Universitario Virgen del Rocio, Av Manuel Siurot s/n, 41013 Sevilla, Spain; franciscoc.munoz.sspa@juntadeandalucia.es

The authors noticed that content of “Conflicts of Interest” in the original version [1] was missing and would like to make a correction:

It should be changed from “Conflicts of Interest: The authors declare no conflict of interest.” to “Conflicts of Interest: Nadia Hindi reports grants, personal fees and non-financial support from PharmaMar, personal fees from Eli Lilly, grants from Eisai, and Novartis, outside the submitted work and research funding for clinical studies (institutional) from PharmaMar, Eli Lilly and Company, AROG, Bayer, Eisai, Lixte, Karyopharm, Deciphera, GlaxoSmithKline, Novartis, Blueprint, Nektar, Forma, Amgen, Bristol Myers Squibb, Pfizer and Daichii-Sankyo. Irene Carrasco García, Alberto Sánchez-Camacho, Johanna Benedetti, Pilar Sancho, and Paloma Santos declare research funding for clinical studies (institutional) from PharmaMar, Eli Lilly and Company, AROG, Bayer, Eisai, Lixte, Karyopharm, Deciphera, GlaxoSmithKline, Novartis, Blueprint, Nektar, Forma, Amgen, and Daichii–Sankyo. Paloma Sanchez-Bustos and David S. Moura report institutional research grants from PharmaMar, Eisai, Immix BioPharma, and Novartis outside the submitted work; travel support from PharmaMar, Eisai, Celgene, Bayer, and Pfizer. Javier Martin-Broto reports research grants from PharmaMar, Eisai, Immix BioPharma, and Novartis outside the submitted work; honoraria for advisory board participation and expert testimony from PharmaMar, Eli Lilly, and Company, Bayer, and Eisai; and research funding for clinical studies (institutional) from PharmaMar, Eli Lilly, and Company, AROG, Bayer, Eisai, Lixte, Karyopharm, Deciphera, GlaxoSmithKline, Novartis, Blueprint, Nektar, Forma, Amgen, Bristol Myers Squibb, Pfizer, and Daichii-Sankyo. All the other authors report no conflicts of interest.”

We apologize for this error and state that the scientific conclusions are unaffected. The original article has been updated.
